# Supplementary material for: Kinetics and mapping of Ca-driven calmodulin conformations on skeletal and cardiac muscle ryanodine receptors
Source: Nat Commun. 2024 Jun 15;15:5120. doi: 10.1038/s41467-024-48951-5 (PMC11180167; doi:10.1038/s41467-024-48951-5)
Supplement: Supplementary file 1 — Supplementary Information [file 41467_2024_48951_MOESM1_ESM.pdf]

SUPPLEMENTARY INFORMATION

Kinetics and Mapping of Ca-driven Calmodulin conformations on Skeletal and Cardiac Muscle Ryanodine Receptors

Robyn T. Rebbeck[1#]*, Bengt Svensson[1#], Jingyan Zhang[1], Montserrat Samsó[2], David D. Thomas[1], Donald M. Bers[3], and Razvan L. Cornea[1#]*

From the [1]Department of Biochemistry, Molecular Biology & Biophysics, University of Minnesota, Minneapolis, MN, USA 55455;
[2]Department of Physiology and Biophysics, Virginia Commonwealth University, Richmond, VA, USA 23298;
[3]Department of Pharmacology, University of California at Davis, Davis, CA, USA 95616.
[#]These authors contributed equally.
*Correspondence:
Robyn T. Rebbeck, Department of Biochemistry, Molecular Biology, and Biophysics, University of Minnesota, 321 Church Street SE, Minneapolis, MN 55455, USA. Tel.: (612) 626-3225; E-mail: rrebbeck@umn.edu

Razvan L. Cornea, Department of Biochemistry, Molecular Biology, and Biophysics, University of Minnesota, 321 Church Street SE, Minneapolis, MN 55455, USA. E-mail: corne002@umn.edu

Supplementary Table 1. Comparison between cryo-EM and FRET-determined distances (used for trilateration) between probe attached to the indicated site (X) on FKBP and N-lobe residues (T26 and T34) on CaM, both bound to RyR1 in skeletal SR membranes. Cryo-EM PDB 6X32 and 7TZC were used to model probe sites on FKBP and CaM, both bound to RyR1, and then distances between probes sites were measured. FLT-FRET was used to model Gaussian distributions between dye probe sites, and the table indicates the values used for trilateration.

RyR1 FKBP-X to CaM-26

	Apo-CaM (closed)	Ca-CaM (closed)								
	6X32	7TZC	CaM nM Ca^{2+}		CaM μM Ca^{2+}		CaM_{1234} nM Ca^{2+}		CaM_{1234} μM Ca^{2+}	
FKBP	Dist.	Dist.	FRET Dist.	FWHM	FRET Dist.	FWHM	FRET Dist.	FWHM	FRET Dist.	FWHM
1	39.0	35.8	52.8	19.8	75.0	17.5	52.0	15.2	52.0	15.7
6	55.0	51.3	57.7	20.8	59.9	31.5	58.2	27.0	71.6	17.8
14	49.5	46.3	56.4	12.6	57.0	18.9	68.5	18.2	62.8	26.9
32	42.8	37.6	45.6	29.6	60.8	48.0	44.6	29.6	45.7	26.8
44	42.5	37.3	54.6	18.8	52.6	21.1	56.3	21.9	57.1	23.5
49	28.1	24.3	49.3	14.4	48.6	20.8	51.3	19.1	51.4	19.9
65	45.8	42.7	55.4	17.8	63.5	27.8	73.6	25.9	52.0	11.1
85	26.7	22.5	38.4	31.7	46.7	57.9	39.2	28.0	40.6	18.5

RyR1 FKBP-X to CaM-34

	Apo-CaM (closed)	Ca-CaM (closed)								
	6X32	7TZC	CaM nM Ca^{2+}		CaM μM Ca^{2+}		CaM_{1234} nM Ca^{2+}		CaM_{1234} μM Ca^{2+}	
FKBP	Dist.	Dist.	FRET Dist.	FWHM	FRET Dist.	FWHM	FRET Dist.	FWHM	FRET Dist.	FWHM
1	52.2	43.0	68.4	24.0	72.6	18.3	66.3	29.6	60.2	20.9
6	64.4	55.7	61.2	19.3	61.1	20.3	58.9	22.2	55.7	10.4
14	52.4	45.4	52.5	15.7	54.0	15.7	51.1	10.6	52.4	15.4
32	62.2	52.5	61.9	50.2	67.8	50.2	58.9	18.1	59.5	16.2
44	49.0	41.6	52.3	18.7	50.8	18.7	55.3	23.8	51.8	12.9
49	33.1	25.2	48.0	19.3	46.0	19.3	46.4	19.7	45.2	21.8
65	55.7	47.0	69.0	22.0	61.3	20.1	57.2	15.0	55.4	8.9
85	46.2	36.8	53.9	24.9	52.3	27.2	49.3	28.0	49.5	25.0

Supplementary Table 2. Comparison between cryo-EM and FRET-determined distances (used for trilateration) between probes attached to the indicated site (X) on FKBP and C-lobe residues (T99 and T110) on CaM, both bound to RyR1 in skeletal SR membranes. Cryo-EM PDB 6X32 and 7TZC were used to model probe sites on FKBP and CaM, both bound to RyR1, and then distances between probes sites were measured. FLT-FRET was used to model Gaussian distributions between dye probe sites, and the table indicates the values used for trilateration.

RyR1 FKBP-X to CaM-99

	Apo-CaM (closed)	Ca-CaM (closed)								
	6X32	7TZC	CaM nM Ca^{2+}		CaM μM Ca^{2+}		CaM_{1234} nM Ca^{2+}		CaM_{1234} μM Ca^{2+}	
FKBP	Dist.	Dist.	FRET Dist.	FWHM	FRET Dist.	FWHM	FRET Dist.	FWHM	FRET Dist.	FWHM
1	100.6	96.6	101.1	27.1	95.7	20.9	102.8	31.3	98.4	35.9
6	110.4	107.0	101.3	21.3	98.8	27.5	111.1	32.6	101.3	28.0
14	92.9	90.2	87.7	14.3	86.9	18.2	92.9	21.0	93.4	14.3
32	102.6	98.0	90.4	12.9	88.6	20.2	92.5	23.4	89.6	16.1
44	78.6	75.4	86.9	9.7	78.9	12.3	86.5	12.9	87.0	21.3
49	71.5	68.1	83.7	11.5	76.9	14.4	86.3	18.7	84.7	17.9
65	103.5	99.9	103.5	20.5	94.2	17.7	106.1	28.3	103.5	29.8
85	89.2	84.7	81.7	10.4	81.9	19.8	88.7	23.2	89.5	25.4

RyR1 FKBP-X to CaM-110

	Apo-CaM (closed)	Ca-CaM (closed)								
	6X32	7TZC	CaM nM Ca^{2+}		CaM μM Ca^{2+}		CaM_{1234} nM Ca^{2+}		CaM_{1234} μM Ca^{2+}	
FKBP	Dist.	Dist.	FRET Dist.	FWHM	FRET Dist.	FWHM	FRET Dist.	FWHM	FRET Dist.	FWHM
1	106.3	103.4	108.9	35.6	104.9	42.9	106.6	33.9	103.6	26.9
6	115.3	111.8	112.5	25.5	96.0	26.4	112.6	23.0	106.0	20.6
14	99.2	95.8	88.0	13.0	80.8	10.6	97.2	20.5	95.2	20.0
32	101.0	97.7	96.6	10.6	81.3	13.2	100.4	21.2	101.2	24.7
44	77.4	73.3	82.1	10.0	74.0	14.4	87.4	11.6	87.1	20.4
49	76.2	72.9	86.8	14.5	74.2	14.2	92.9	22.8	91.5	18.5
65	109.7	106.6	106.8	20.6	90.9	17.5	111.9	28.8	108.4	28.5
85	99.4	88.7	87.8	17.4	81.1	29.0	95.7	19.4	94.6	26.2

Supplementary Table 3. Comparison between cryo-EM and FRET-determined distances (used for trilateration) between probes attached to the indicated site (X) on FKBP and N-lobe residues (T26 and T34) on CaM, both bound to RyR2 in cardiac SR membranes. Cryo-EM PDB 6JI8 and 6JV2 were used to model probe sites on FKBP and CaM, both bound to RyR2, and then distances between probes sites were measured. FLT-FRET was used to model Gaussian distributions between dye probe sites, and the table indicates the values used for trilateration.

RyR2 FKBP-X to CaM-26

	Apo-CaM (closed)	Ca-CaM (closed)								
	6JI8	6JV2	CaM nM Ca^{2+}		CaM μM Ca^{2+}		CaM_{1234} nM Ca^{2+}		CaM_{1234} μM Ca^{2+}	
FKBP	Dist.	Dist.	FRET Dist.	FWHM	FRET Dist.	FWHM	FRET Dist.	FWHM	FRET Dist.	FWHM
1	46.0	76.1	52.0	9.7	57.9	13.2	52.4	12.0	51.7	9.3
6	58.5	84.6	55.3	10.5	54.6	19.9	60.6	15.2	60.3	15.7
14	56.1	74.2	54.0	10.7	52.7	35.1	68.3	19.3	63.1	29.2
32	44.5	73.9	57.2	42.9	57.4	48.2	44.7	32.9	45.3	21.6
44	43.6	57.8	38.0	36.4	42.4	31.8	52.9	21.0	52.3	21.8
49	31.1	48.4	45.0	20.3	50.7	22.4	48.4	17.7	44.7	26.1
65	52.4	79.0	59.9	15.9	61.0	18.6	61.2	16.9	61.6	15.7
85	27.6	57.7	32.2	35.1	50.2	36.9	36.6	34.8	41.6	22.7

RyR2 FKBP-X to CaM-34

	Apo-CaM (closed)	Ca-CaM (closed)								
	6JI8	6JV2	CaM nM Ca^{2+}		CaM μM Ca^{2+}		CaM_{1234} nM Ca^{2+}		CaM_{1234} μM Ca^{2+}	
FKBP	Dist.	Dist.	FRET Dist.	FWHM	FRET Dist.	FWHM	FRET Dist.	FWHM	FRET Dist.	FWHM
1	58.6	95.9	63.3	9.6	59.7	12.9	56.5	13.5	60.8	9.9
6	66.9	106.1	62.8	10.0	60.4	16.7	63.9	13.4	63.4	19.3
14	57.0	96.0	54.5	31.3	58.6	32.4	50.6	14.2	52.4	18.8
32	62.9	90.4	104.2	51.6	74.9	33.2	62.8	22.5	64.7	25.1
44	47.4	75.6	51.2	26.8	52.6	24.6	52.9	22.6	49.1	15.6
49	32.8	70.0	50.7	28.8	46.8	40.5	44.4	17.5	39.2	28.2
65	61.0	100.7	62.4	7.3	62.7	18.0	62.4	9.7	62.2	9.3
85	46.7	75.2	51.0	15.4	51.4	39.4	48.5	26.4	51.0	20.1

Supplementary Table 4. Comparison between cryo-EM and FRET-determined distances (used for trilateration) between probes attached to the indicated site (X) on FKBP and C-lobe residues (T99 and T110) on CaM, both bound to RyR2 in cardiac SR membranes. Cryo-EM PDB 6JI8 and 6JV2 were used to model probe sites on FKBP and CaM, both bound to RyR2, and then distances between probes sites were measured. FLT-FRET was used to model Gaussian distributions between dye probe sites, and the table indicates the values used for trilateration.

RyR2 FKBP-X to CaM-99

FKBP	Apo-CaM (closed) 6JI8 Dist.	Ca-CaM (closed) 6JV2 Dist.	CaM nM Ca^{2+} FRET Dist.	FWHM	CaM μM Ca^{2+} FRET Dist.	FWHM	CaM_{1234} nM Ca^{2+} FRET Dist.	FWHM	CaM_{1234} μM Ca^{2+} FRET Dist.	FWHM
1	106.3	123.5	115.8	61.6	101.1	46.4	104.9	46.7	114.8	57.5
6	112.9	128.3	110.8	40.8	97.9	27.5	93.1	30.8	96.5	24.8
14	97.3	110.3	86.0	24.2	81.6	17.3	95.1	27.4	96.1	23.1
32	104.2	121.9	101.8	5.0	94.2	5.0	93.4	25.3	86.3	24.6
44	78.2	91.4	91.0	30.6	85.9	22.6	86.2	19.7	84.3	15.3
49	74.0	89.1	90.6	13.0	88.8	17.8	83.9	23.5	86.7	25.1
65	108.2	124.1	108.6	37.3	101.6	34.8	95.8	33.3	86.2	24.5
85	89.4	107.7	96.2	34.8	94.9	32.4	86.2	15.8	90.1	34.2

RyR2 FKBP-X to CaM-110

FKBP	Apo-CaM (closed) 6JI8 Dist.	Ca-CaM (closed) 6JV2 Dist.	CaM nM Ca^{2+} FRET Dist.	FWHM	CaM μM Ca^{2+} FRET Dist.	FWHM	CaM_{1234} nM Ca^{2+} FRET Dist.	FWHM	CaM_{1234} μM Ca^{2+} FRET Dist.	FWHM
1	106.2	114.0	119.6	62.8	104.2	51.7	119.8	62.1	116.5	61.3
6	112.7	121.1	110.8	23.2	94.8	24.6	91.9	20.8	98.4	25.7
14	99.1	107.0	92.9	34.5	84.2	24.8	96.7	24.1	98.1	22.2
32	98.4	107.5	107.6	5.0	89.3	35.0	100.5	37.2	101.8	37.5
44	74.2	83.6	87.8	30.9	77.9	21.8	85.0	18.9	87.6	21.6
49	76.3	83.5	94.4	5.7	84.1	17.2	89.0	21.9	94.9	20.2
65	109.0	116.9	111.8	36.7	98.6	32.0	94.4	26.1	94.9	35.4
85	86.7	94.5	100.5	31.6	92.3	39.0	89.3	13.9	85.2	18.5

Supplementary Table 5. Comparison between cryo-EM and trilateration loci (from FRET data) distances between probes attached to the indicated site (X) on FKBP and N-lobe residues (T26 and T34) on CaM, both bound to RyR1 in skeletal SR membranes. Cryo-EM PDB 6X32 and 7TZC were used to model probe sites on FKBP and CaM, both bound to RyR1, and then distances between probes sites were measured.

RyR1 FKBP-X to CaM-26

FKBP	Apo-CaM (closed) 6X32 Dist.	Ca-CaM (closed) 7TZC Dist.	CaM nM Ca^{2+} Locus Dist.	CaM μCa^{2+} Locus Dist.	CaM_{1234} nM Ca^{2+} Locus Dist.	CaM_{1234} μM Ca^{2+} Locus Dist.
1	39.0	35.8	47.3	65.1	52.8	49.1
6	55.0	51.3	64.2	76.6	71.5	67.2
14	49.5	46.3	58.2	62.6	67.8	62.6
32	42.8	37.6	53.2	68.0	51.7	53.7
44	42.5	37.3	53.6	49.9	59.0	58.0
49	28.1	24.3	38.1	39.1	45.9	42.7
65	45.8	42.7	54.4	68.9	61.8	57.1
85	26.7	22.5	36.5	53.9	36.5	37.2

RyR1 FKBP-X to CaM-34

FKBP	Apo-CaM (closed) 6X32 Dist.	Ca-CaM (closed) 7TZC Dist.	CaM nM Ca^{2+} Locus Dist.	CaM μCa^{2+} Locus Dist.	CaM_{1234} nM Ca^{2+} Locus Dist.	CaM_{1234} μM Ca^{2+} Locus Dist.
1	52.2	43.0	59.8	56.7	52.3	50.5
6	64.4	55.7	71.4	69.1	64.4	64.6
14	52.4	45.4	57.8	56.5	51.9	54.8
32	62.2	52.5	66.5	64.0	59.6	58.4
44	49.0	41.6	49.7	49.6	44.7	50.0
49	33.1	25.2	36.1	35.2	30.1	34.3
65	55.7	47.0	63.3	60.6	56.1	55.5
85	46.2	36.8	51.2	48.4	44.3	42.1

Supplementary Table 6. Comparison between cryo-EM and trilateration loci (from FRET data) distances between probes attached to indicated site (X) on FKBP and N-lobe residues (T99 and T110) on CaM, both bound to RyR1 in skeletal SR membranes. Cryo-EM PDB 6X32 and 7TZC were used to model probe sites on FKBP and CaM, both bound to RyR1, and then distances between probes sites were measured.

RyR1 FKBP-X to CaM-99

FKBP	Apo-CaM (closed) 6X32 Dist.	Ca-CaM (closed) 7TZC Dist.	CaM nM Ca^{2+} Locus Dist.	CaM μCa^{2+} Locus Dist.	CaM_{1234} nM Ca^{2+} Locus Dist.	CaM_{1234} μM Ca^{2+} Locus Dist.
1	100.6	96.6	96.5	93.3	102.7	95.3
6	110.4	107.0	108.6	106.8	116.1	108.8
14	92.9	90.2	94.8	93.5	103.0	95.9
32	102.6	98.0	92.0	91.4	98.4	92.1
44	78.6	75.4	75.5	77.7	85.0	79.0
49	71.5	68.1	70.3	69.4	78.4	71.4
65	103.5	99.9	101.6	98.8	108.5	101.0
85	89.2	84.7	80.6	78.2	86.3	79.4

RyR1 FKBP-X to CaM-110

FKBP	Apo-CaM (closed) 6X32 Dist.	Ca-CaM (closed) 7TZC Dist.	CaM nM Ca^{2+} Locus Dist.	CaM μCa^{2+} Locus Dist.	CaM_{1234} nM Ca^{2+} Locus Dist.	CaM_{1234} μM Ca^{2+} Locus Dist.
1	106.3	103.4	101.8	83.6	106.8	103.0
6	115.3	111.8	114.4	96.4	118.7	114.1
14	99.2	95.8	100.8	82.7	103.8	98.1
32	101.0	97.7	96.9	84.3	103.9	102.4
44	77.4	73.3	81.6	69.0	86.1	81.8
49	76.2	72.9	76.2	59.2	80.0	75.2
65	109.7	106.6	107.2	88.4	111.5	107.0
85	99.4	88.7	85.5	70.3	91.7	89.6

Supplementary Table 7. Comparison between cryo-EM and trilateration loci (from FRET data) distances between probes attached to the indicated site (X) on FKBP and N-lobe residues (T26 and T34) on CaM, both bound to RyR2 in cardiac SR membranes. Cryo-EM PDB 6JI8 and 6JV2 were used to model probe sites on FKBP and CaM, both bound to RyR1, and then distances between probes sites were measured.

RyR2 FKBP-X to CaM-26

FKBP	Apo-CaM (closed) 6JI8 Dist.	Ca-CaM (closed) 6JV2 Dist.	CaM nM Ca^{2+} Locus Dist.	CaM μCa^{2+} Locus Dist.	CaM_{1234} nM Ca^{2+} Locus Dist.	CaM_{1234} μM Ca^{2+} Locus Dist.
1	46.0	76.1	50.5	57.0	52.3	51.3
6	58.5	84.6	60.0	64.2	66.1	64.4
14	56.1	74.2	52.8	52.8	64.6	62.1
32	44.5	73.9	51.3	60.4	51.3	51.2
44	43.6	57.8	39.5	40.6	53.8	51.5
49	31.1	48.4	26.7	27.7	40.2	37.5
65	52.4	79.0	54.3	58.8	59.5	57.9
85	27.6	57.7	35.4	45.1	34.0	33.8

RyR2 FKBP-X to CaM-34

FKBP	Apo-CaM (closed) 6JI8 Dist.	Ca-CaM (closed) 6JV2 Dist.	CaM nM Ca^{2+} Locus Dist.	CaM μCa^{2+} Locus Dist.	CaM_{1234} nM Ca^{2+} Locus Dist.	CaM_{1234} μM Ca^{2+} Locus Dist.
1	58.6	95.9	60.8	58.7	57.2	60.0
6	66.9	106.1	66.4	67.6	66.3	67.7
14	57.0	96.0	52.7	58.5	57.8	56.8
32	62.9	90.4	63.6	63.1	61.6	63.9
44	47.4	75.6	38.4	49.4	48.9	46.0
49	32.8	70.0	28.1	34.4	33.7	32.3
65	61.0	100.7	61.5	61.5	60.2	62.0
85	46.7	75.2	49.2	46.7	45.1	48.0

Supplementary Table 8. Comparison between cryo-EM and trilateration loci (from FRET data) distances between probes attached to the indicated site (X) on FKBP and N-lobe residues (T99 and T110) on CaM, both bound to RyR2 in cardiac SR membranes. Cryo-EM PDB 6JI8 and 6JV2 were used to model probe sites on FKBP and CaM, both bound to RyR1, and then distances between probes sites were measured.

RyR2 FKBP-X to CaM-99

FKBP	Apo-CaM (closed) 6JI8 Dist.	Ca-CaM (closed) 6JV2 Dist.	CaM nM Ca^{2+} Locus Dist.	CaM μCa^{2+} Locus Dist.	CaM_{1234} nM Ca^{2+} Locus Dist.	CaM_{1234} μM Ca^{2+} Locus Dist.
1	106.3	123.5	104.2	94.6	104.5	101.1
6	112.9	128.3	111.0	99.7	113.9	111.1
14	97.3	110.3	96.1	83.1	102.3	100.2
32	104.2	121.9	100.7	93.5	99.6	96.3
44	78.2	91.4	75.3	64.0	81.9	80.4
49	74.0	89.1	72.4	60.6	76.8	74.5
65	108.2	124.1	106.5	95.4	108.7	105.7
85	89.4	107.7	86.4	79.4	84.5	81.0

RyR2 FKBP-X to CaM-110

FKBP	Apo-CaM (closed) 6JI8 Dist.	Ca-CaM (closed) 6JV2 Dist.	CaM nM Ca^{2+} Locus Dist.	CaM μCa^{2+} Locus Dist.	CaM_{1234} nM Ca^{2+} Locus Dist.	CaM_{1234} μM Ca^{2+} Locus Dist.
1	106.2	114.0	109.8	94.5	108.0	103.3
6	112.7	121.1	115.5	102.4	116.5	112.3
14	99.1	107.0	99.1	88.7	103.6	99.8
32	98.4	107.5	107.0	93.9	103.5	100.1
44	74.2	83.6	78.7	72.0	83.1	80.9
49	76.3	83.5	76.6	64.4	78.8	74.7
65	109.0	116.9	111.3	97.1	111.6	107.0
85	86.7	94.5	93.0	78.1	88.6	84.5

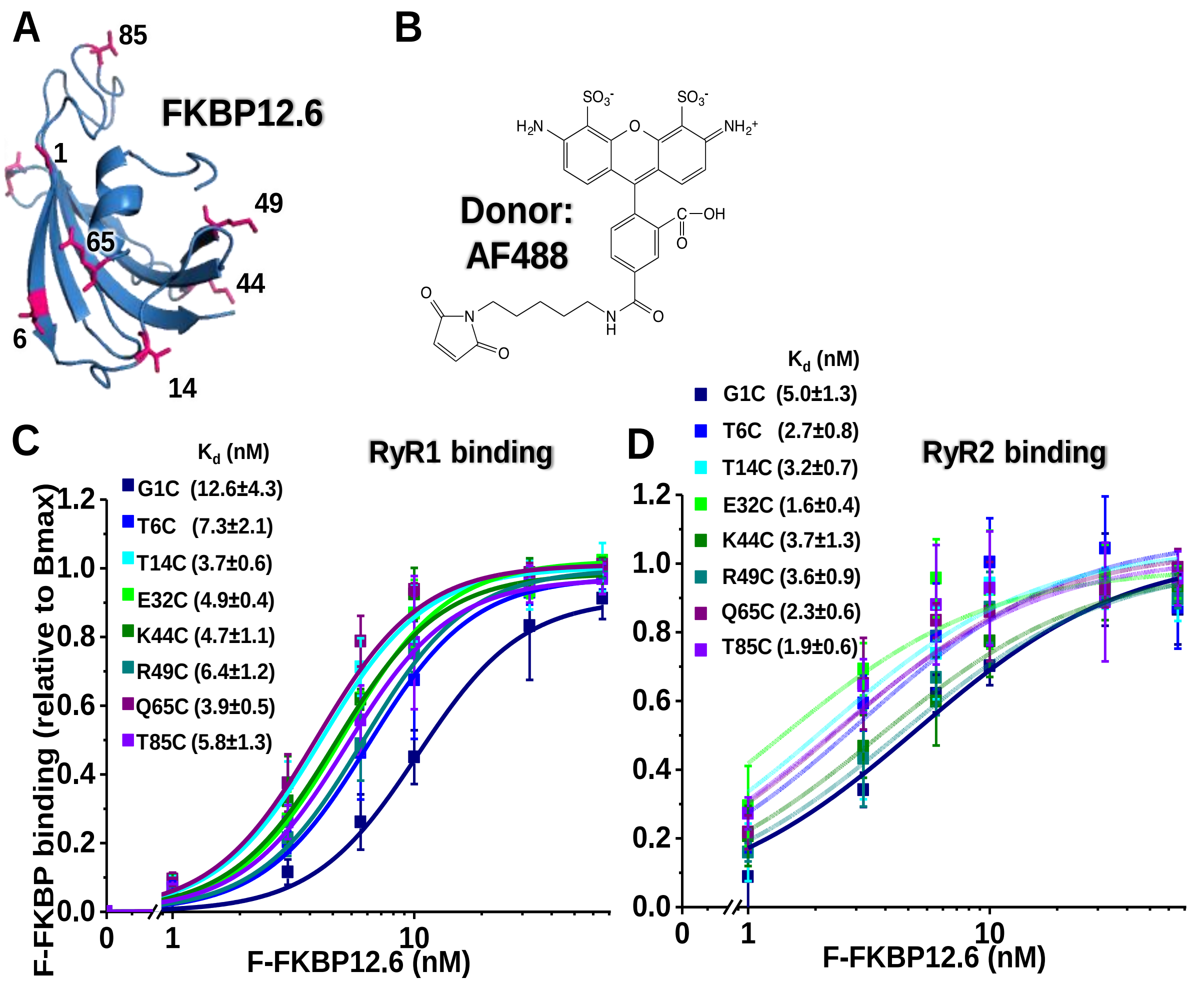

Supplementary Fig. 1 Eight labeling sites on FKBP12.6 and impact on binding to RyR. **A**) Crystal structure of FKBP12.6 (blue) with sites mutated to Cys for AF488 C5 maleimide labeling shown in pink. **B**) Chemical structure of AF488 C5 maleimide. **C** and **D**) Fluorescence intensity indicative of D-FKBP binding to skeletal (**C**) and cardiac (**D**) SR following sedimentation to remove unbound D-FKBP. FKBP label site data shown as dark blue for site 1, blue for site 6, light blue for site 14, light green for site 32, dark green for site 44, teal for site 49, purple for site 65 and light purple for site 85. Data shown as mean ±SD, n =3 (**C**) and 5 (**D**) experiments undertaken on separate days. K_d values shown in parenthesis from Hill fit to data using Origin 2015 software.

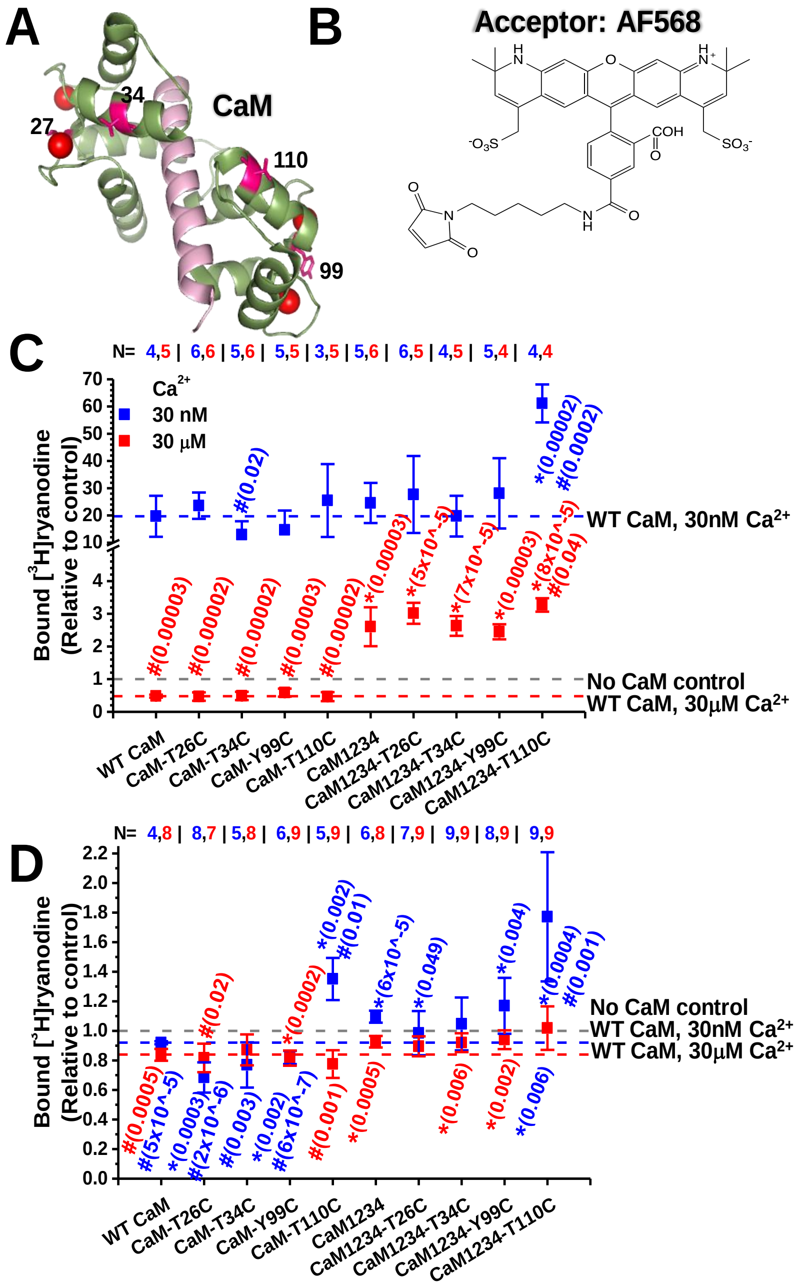

Supplementary Fig. 2 Four labeling sites on Ca^{2+}-sensitive and -insensitive CaM and functional impact of labeling on binding to RyR. A) Crystal structure of CaM with labeling sites (pink) for shown FRET donor probe AF568. **B**) Chemical structure of AF568 C5 maleimide. C and D) SR membranes from skeletal (**C**) or cardiac (**D**) muscle were incubated with indicated CaM (0 or 800 nM) at 30 nM (blue) or 30 μM (red) Ca^{2+} in the presence of [^{3}H]ryanodine. The fraction of [^{3}H]ryanodine binding is relative to "no CaM" control indicated with gray line, with the WT CaM effect at 30nM and 30 μM Ca^{2+} indicated with blue and red dotted lines, respectively. Data shown as mean ±SD, with the number of independent experiments from separate days indicated above each data set (blue for nM Ca^{2+} and red for μM Ca^{2+}). *Significant differences relative to WT-CaM control, p<0.05, p value in parenthesis. $^{\#}$Significant differences relative to Ca-insensitive CaM (CaM_{1234}), p<0.05. Significance was determined using unpaired, two-way Student's T-test.

RyR1 Gaussian Fit parameters

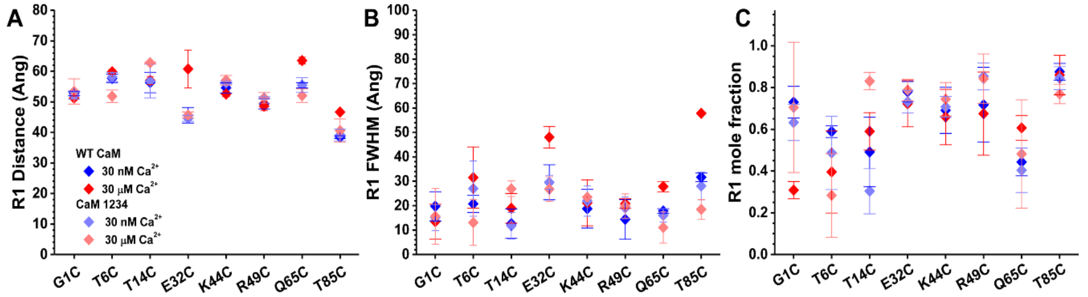

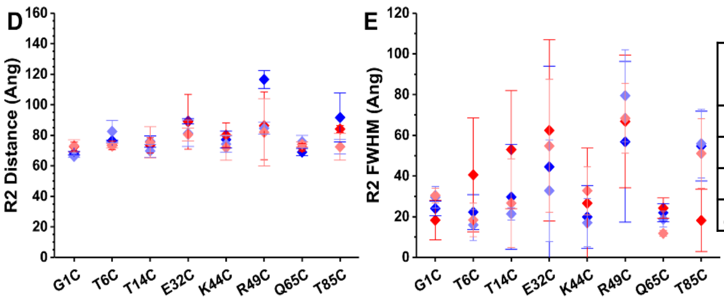

Independent repeat #	D-X-FKBP							
	1	6	14	32	44	49	65	85
WT CaM, 30 nM Ca^{2+}	3	3	4	3	4	4	3	3
WT CaM, 30 mM Ca^{2+}	3	3	4	3	4	4	3	3
CaM1234, 30 nM Ca^{2+}	3	3	3	3	3	4	3	3
CaM1234, 30 mM Ca^{2+}	3	3	3	4	3	4	3	4

RyR2 Gaussian Fit parameters

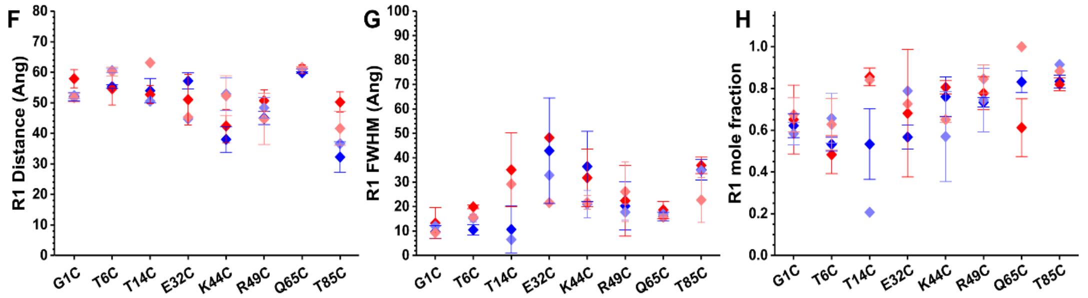

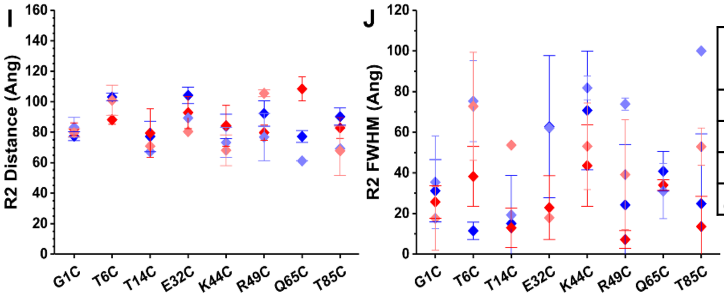

Independent repeat #	D-X-FKBP							
	1	6	14	32	44	49	65	85
WT CaM, 30 nM Ca^{2+}	3	3	4	3	4	4	3	3
WT CaM, 30 mM Ca^{2+}	3	3	4	3	4	4	3	3
CaM1234, 30 nM Ca^{2+}	3	3	1	1	2	2	3	1
CaM1234, 30 mM Ca^{2+}	3	3	1	1	2	2	3	2

Supplementary Fig. 3 Gaussian fit parameters for AF568-T26C-CaM. SR membranes from porcine skeletal (A-E) or cardiac (F-J) muscle were labeled with D-FKBP (AF488-X-FKBP; X = Cys mutation site for fluorescence labeling), and then incubated with 800 nM CaM labeled with acceptor probe at the N-lobe residue T26C. FRET assays used CaM (dark) or CaM_{1234} (light) forms of CaM, and contained 30 nM (blue symbols) or 30 µM Ca^{2+} (red symbols). Multi-exponential analysis of FLT-FRET data yielded a two–distance Gaussian distribution model for the separation between D-FKBP and A-CaM within RyR. **A** and **F**) shorter distance (R1), **B** and **G**) full-width half max (FWHM) for R1, **C** and **H**) mole fraction occupied by R1, **D** and **I**) longer distance (R2), and **E** and **J**) FWHM for R2. Data shown as mean ±SD. Number of independent experiments from separate days are shown right of panel **E** for RyR1 and right of panel **J** for RyR2.

RyR1 Gaussian Fit parameters

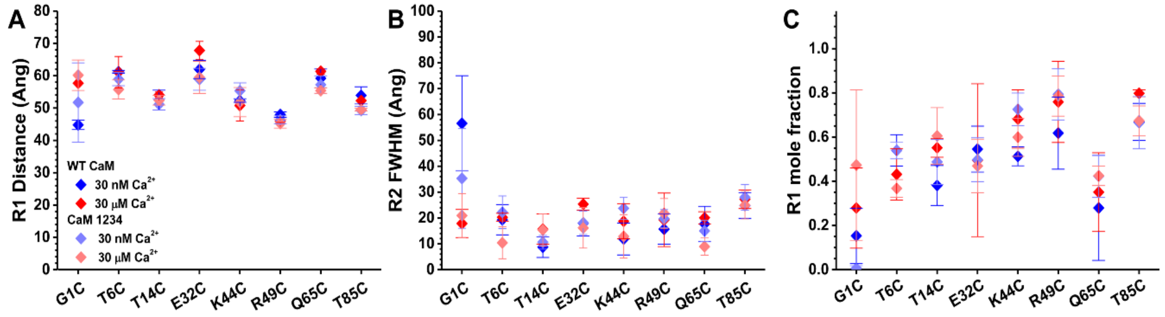

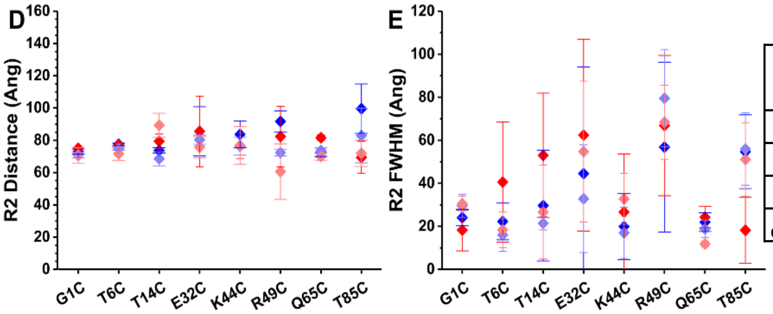

Independent repeat #	D-X-FKBP							
	1	6	14	32	44	49	65	85
WT CaM, 30 nM Ca^{2+}	3	3	3	3	3	4	3	3
WT CaM, 30 mM Ca^{2+}	3	3	4	3	4	4	3	3
CaM1234, 30 nM Ca^{2+}	3	3	3	3	3	4	3	4
CaM1234, 30 mM Ca^{2+}	3	3	3	4	3	4	3	4

RyR2 Gaussian Fit parameters

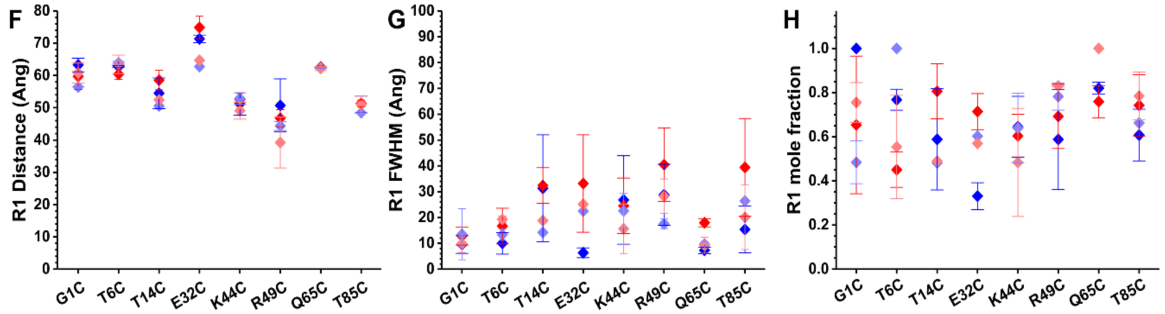

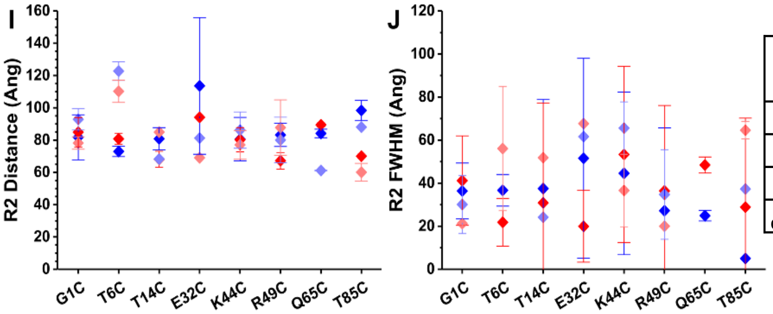

Independent repeat #	D-X-FKBP							
	1	6	14	32	44	49	65	85
WT CaM, 30 nM Ca^{2+}	3	3	4	3	4	3	3	3
WT CaM, 30 mM Ca^{2+}	3	3	4	2	4	4	3	3
CaM1234, 30 nM Ca^{2+}	3	3	1	1	3	2	3	1
CaM1234, 30 mM Ca^{2+}	3	3	1	1	3	2	3	3

Supplementary Fig. 4 Gaussian fit parameters for AF568-T34C-CaM. SR membranes from porcine skeletal (A-E) or cardiac (F-J) muscle were labeled with D-FKBP (AF488-X-FKBP; X = Cys mutation site for fluorescence labeling), and then incubated with 800 nM CaM labeled with acceptor probe at the N-lobe residue T34C. FRET assays used CaM (dark) or CaM_{1234} (light) forms of CaM, and contained 30 nM (blue symbols) or 30 μM Ca^{2+} (red symbols). Multi-exponential analysis of FLT-FRET data yielded a two–distance Gaussian distribution model for the separation between D-FKBP and A-CaM within RyR. **A** and **F**) shorter distance (R1), **B** and **G**) full-width half max (FWHM) for R1, **C** and **H**) mole fraction occupied by R1, **D** and **I**) longer distance (R2), and **E** and **J**) FWHM for R2. Data shown as mean ±SD. Number of independent experiments from separate days are shown right of panel **E** for RyR1 and right of panel **J** for RyR2.

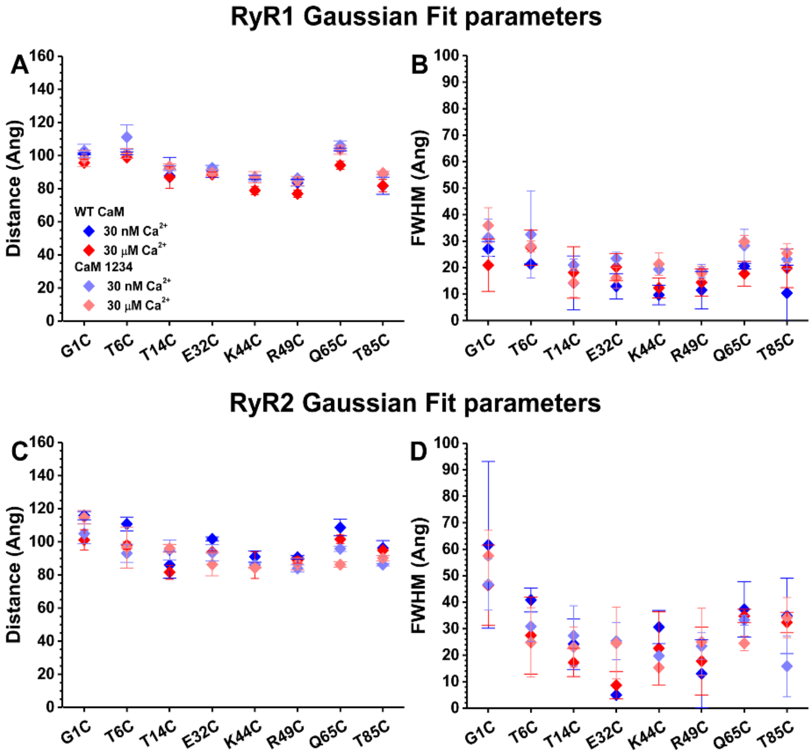

Independent repeat #	D-X-FKBP							
	1	6	14	32	44	49	65	85
WT CaM, 30 nM Ca^{2+}	3	3	4	3	3	4	3	3
WT CaM, 30 mM Ca^{2+}	3	3	3	3	4	4	3	3
CaM1234, 30 nM Ca^{2+}	3	3	3	4	2	4	3	4
CaM1234, 30 mM Ca^{2+}	3	3	3	3	2	4	3	4

Independent repeat #	D-X-FKBP							
	1	6	14	32	44	49	65	85
WT CaM, 30 nM Ca^{2+}	3	3	3	1	4	4	3	3
WT CaM, 30 mM Ca^{2+}	3	3	3	2	4	4	3	3
CaM1234, 30 nM Ca^{2+}	3	3	3	3	2	4	3	4
CaM1234, 30 mM Ca^{2+}	3	3	3	4	1	2	3	2

Supplementary Fig. 5 Gaussian fit parameters for AF568-Y99C-CaM. SR membranes from porcine skeletal (**A** and **B**) or cardiac (**C** and **D**) muscle were labeled with D-FKBP (AF488-X-FKBP; X = Cys mutation site for fluorescence labeling), and then incubated with 800 nM CaM labeled with acceptor probe at the C-lobe residue Y99C. FRET assays used CaM (dark) or CaM_{1234} (light) forms of CaM, and contained 30 nM (blue symbols) or 30 µM Ca^{2+} (red symbols). Multi-exponential analysis of FLT-FRET data yielded a one–distance Gaussian distribution model for the separation between D-FKBP and A-CaM within RyR. **A** and **C**) distance, and **B** and **D**) full-width half max (FWHM) for distance. Data shown as mean ±SD. Number of independent experiments from separate days are shown in right of panel **B** for RyR1 and right of panel **D** for RyR2.

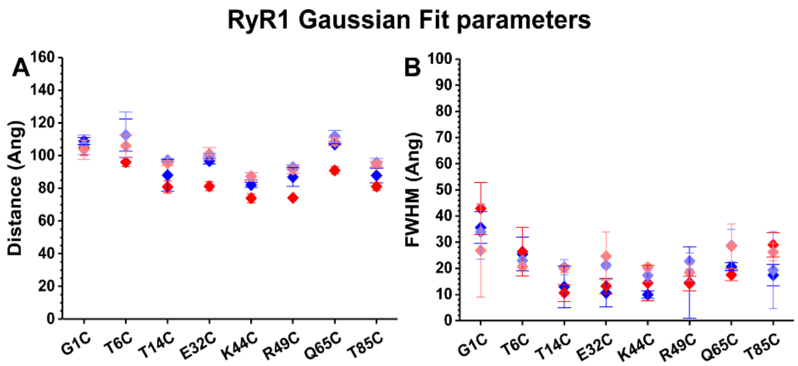

Independent repeat #	D-X-FKBP							
	1	6	14	32	44	49	65	85
WT CaM, 30 nM Ca^{2+}	3	3	4	3	4	4	3	3
WT CaM, 30 mM Ca^{2+}	3	3	3	3	4	4	3	3
CaM1234, 30 nM Ca^{2+}	3	3	3	3	2	4	3	4
CaM1234, 30 mM Ca^{2+}	3	3	3	3	2	4	3	4

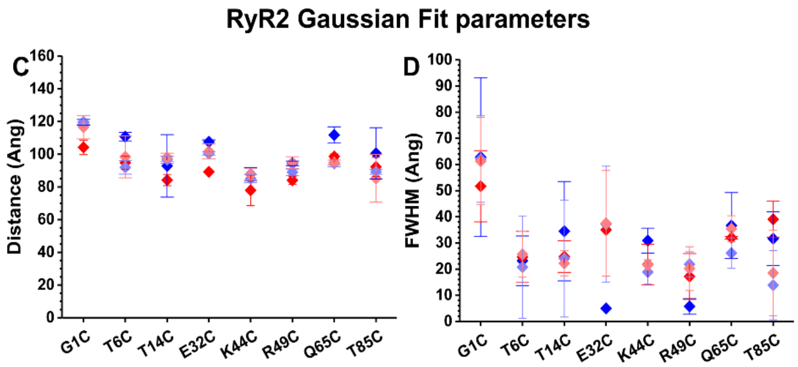

Independent repeat #	D-X-FKBP							
	1	6	14	32	44	49	65	85
WT CaM, 30 nM Ca^{2+}	3	3	3	1	4	4	3	3
WT CaM, 30 mM Ca^{2+}	3	3	3	1	4	4	3	3
CaM1234, 30 nM Ca^{2+}	3	3	3	2	3	4	3	3
CaM1234, 30 mM Ca^{2+}	3	3	3	3	3	3	3	3

Supplementary Fig. 6 Gaussian fit parameters for AF568-T110C-CaM. SR membranes from porcine skeletal (**A** and **B**) or cardiac (**C** and **D**) muscle were labeled with D-FKBP (AF488-X-FKBP; X = Cys mutation site for fluorescence labeling), and then incubated with 800 nM CaM labeled with acceptor probe at the C-lobe residue T110C. FRET assays used CaM (dark) or CaM_{1234} (light) forms of CaM, and contained 30 nM (blue symbols) or 30 μM Ca^{2+} (red symbols). Multi-exponential analysis of FLT-FRET data yielded a one–distance Gaussian distribution model for the separation between D-FKBP and A-CaM within RyR. **A** and **C**) distance, and **B** and **D**) full-width half max (FWHM) for distance. Data shown as mean ±SD. Number of independent experiments from separate days are shown right of panel **B** for RyR1 and right of panel **D** for RyR2.

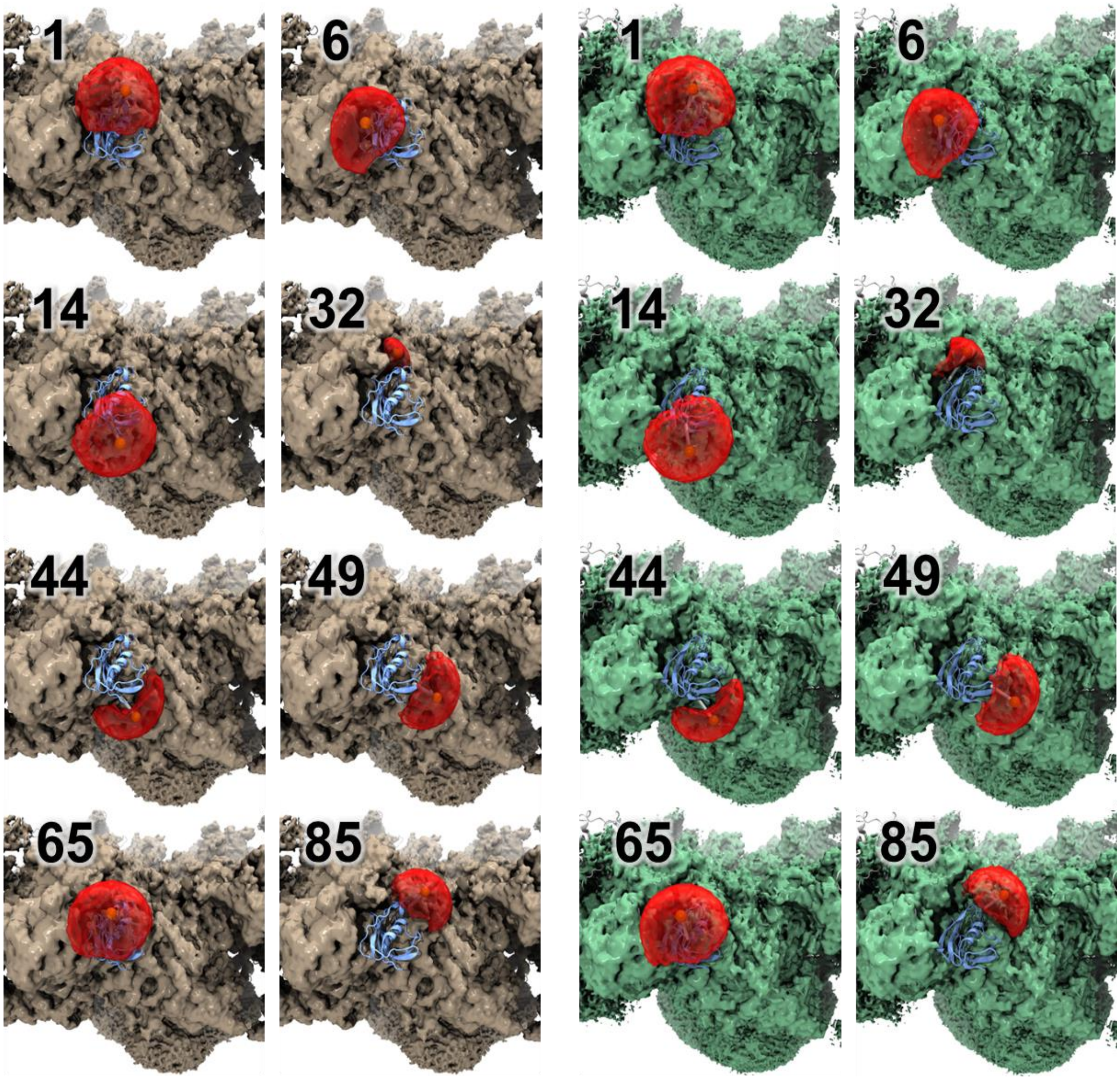

Supplementary Fig. 7 Donor probe location on FKBP bound to RyR1 and RyR2, determined by simulated annealing. The cryo-EM density map of RyR1 is shown in tan color. The cryo-EM density map of RyR2 is shown in green. FKBP is shown in blue. The volume sampled by AF488 attached at specific FKBP sequence positions, as indicated, is shown in red. The calculated average position of the probes that is used as the effective probe location in the trilateration calculations are show as orange spheres within the red volumes. In all cases the probe samples a large volume to allow isotropic motion, except for the probe at position 32 where the space is more restricted.

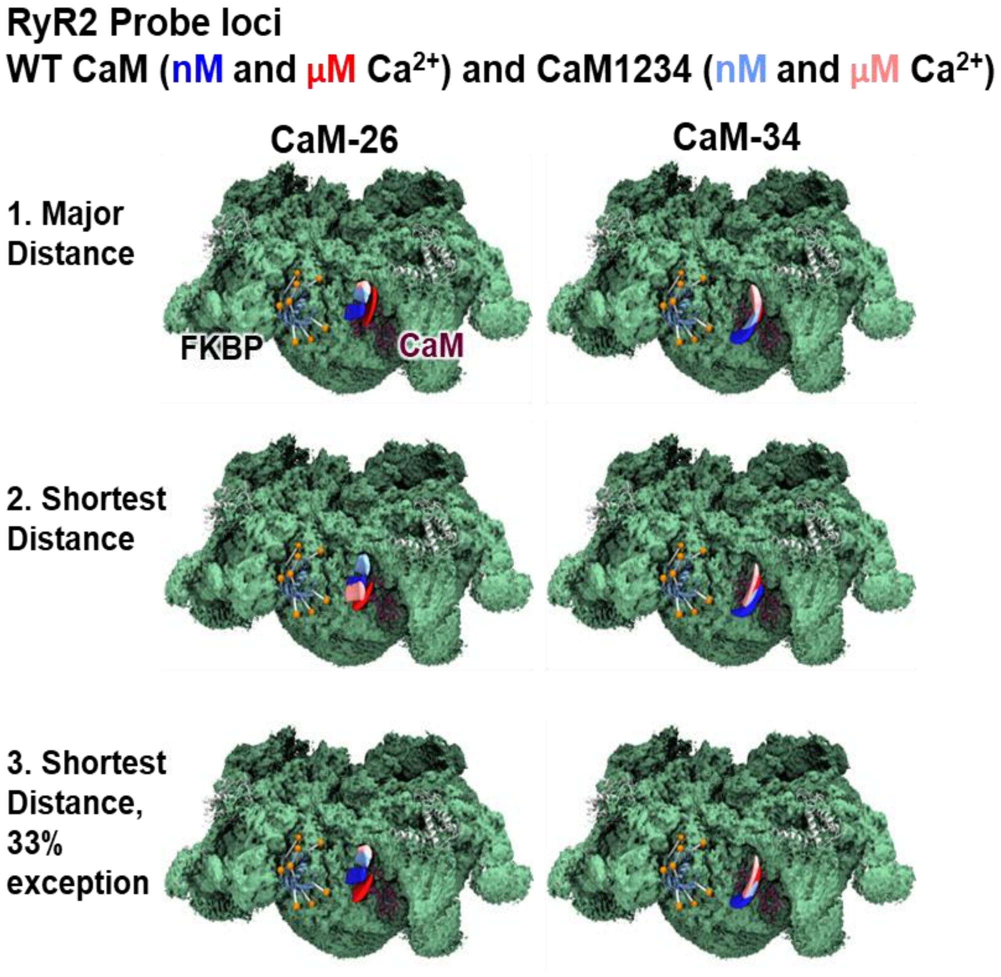

Supplementary Fig. 8 Acceptor probe loci on RyR2 based on using different criteria for distance selection. From the two-distance Gaussian fit to the FRET data, acceptor probe location could be selected from one of two distances. Three different sets of trilateration maps were generated based on criteria for selecting distances that were used for trilateration. This included trilateration with **1**) the distance with the highest mole fraction (major distance), **2**) the shortest distance, and **3**) the shortest distance with the exception for when the longer distance had a mole fraction >66%. The latter method was selected as it avoided the case when two distance populations had about equal weight and either the longer or shorter distance was picked in an arbitrary manner. Depending on which selection one uses, differences in the loci are observed. However, method 3 gave the most consistent result with the CaM_{1234} at low and high Ca^{2+} overlapping in location. The cryo-EM density map of RyR2[4] is shown in green. FKBP is shown as ribbon representation in blue with the donor probe positions shown as orange spheres. Trilaterated loci for AF568 probes bound to indicated CaM residues in assay conditions containing 30 nM and 30 μM free Ca^{2+} are blue and red, respectively. Trilaterated loci for probes bound to Ca^{2+} insensitive CaM (CaM_{1234}) in assay conditions containing 30 nM and 30 μM free Ca^{2+} are light blue and light red, respectively. The maps of RyR2 are shown at 60° from the cytoplasmic face.

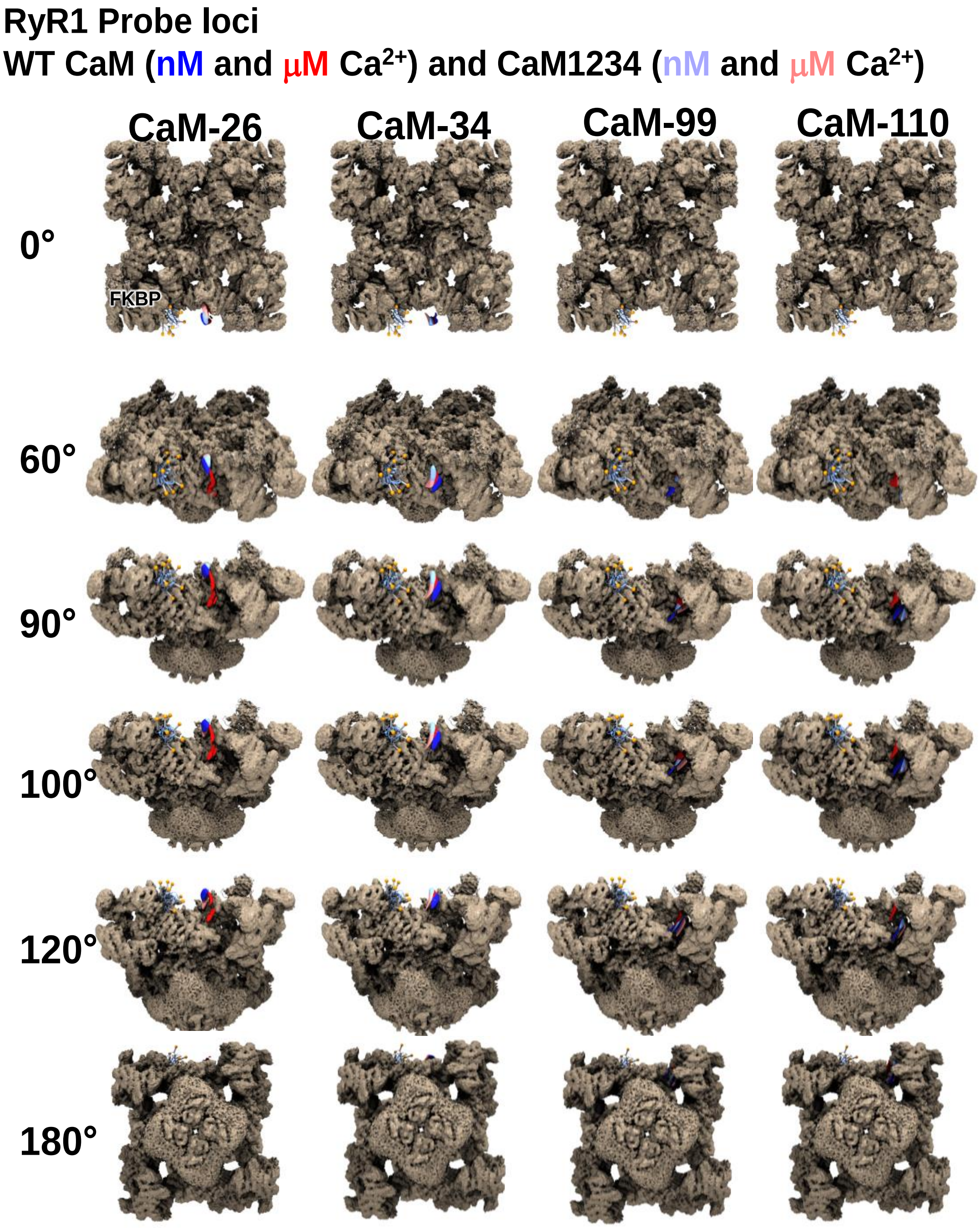

Supplementary Fig. 9 Acceptor probe loci bound to CaM at 26, 34, 99 and 110 on RyR1. The cryo-EM density map of RyR1 is shown in brown. FKBP is shown as ribbon representation in blue with the donor probe positions shown as orange spheres. Trilaterated loci for AF568 probes bound to indicated CaM residues in assay conditions containing 30 nM and 30 μM free Ca^{2+} are blue and red, respectively. Trilaterated loci for probes bound to Ca^{2+} insensitive CaM (CaM_{1234}) in assay conditions containing 30 nM and 30 μM free Ca^{2+} are light blue and light red, respectively. The maps of RyR are shown at indicated degree rotated from the membrane plane.

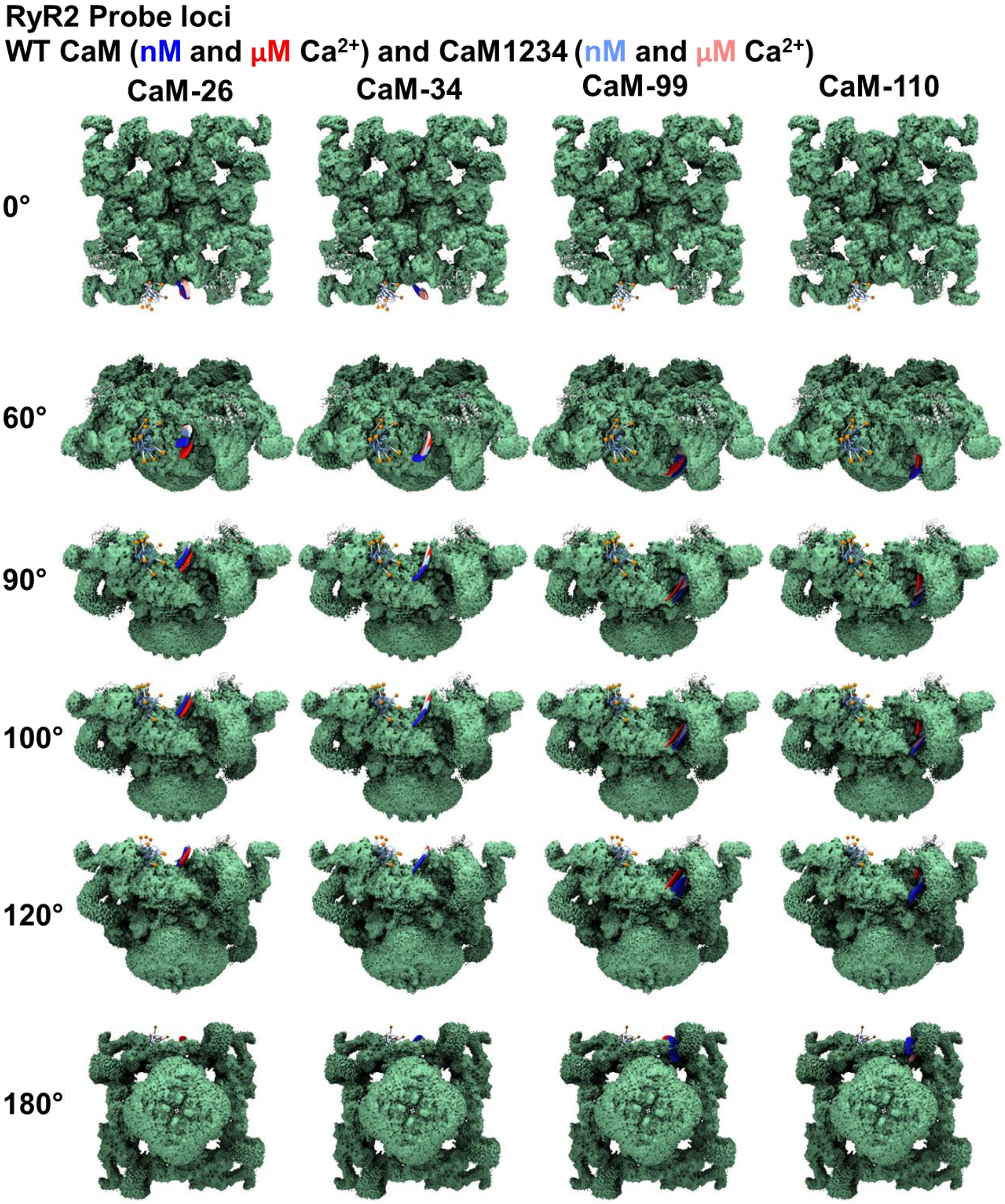

Supplementary Fig. 10 Acceptor probe loci bound to CaM at 26, 34, 99 and 110 on RyR2. The cryo-EM density map of RyR2 is shown in green. FKBP is shown as ribbon representation in blue with the donor probe positions shown as orange spheres. Trilaterated loci for AF568 probes bound to indicated CaM residues in assay conditions containing 30 nM and 30 μM free Ca^{2+} are blue and red, respectively. Trilaterated loci for probes bound to Ca^{2+} insensitive CaM (CaM_{1234}) in assay conditions containing 30 nM and 30 μM free Ca^{2+} are light blue and light red, respectively. The maps of RyR are shown at indicated degree rotated from the membrane plane.

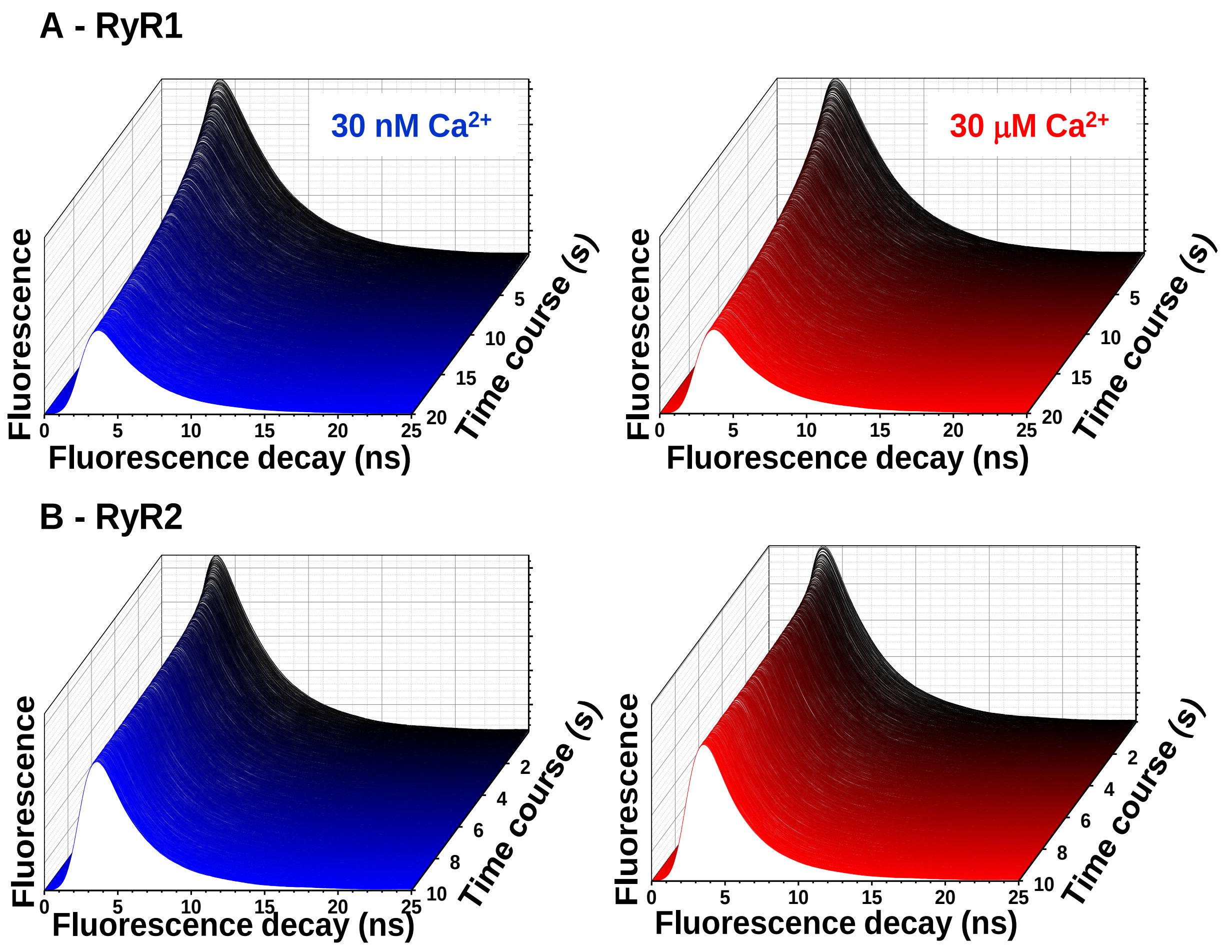

Supplementary Fig. 11 Representative fluorescence decay time course for CaM binding to RyR1 and RyR2 in nM and μM Ca^{2+}. SR membranes from porcine skeletal (**A**) or cardiac (**B**) muscle were labeled with D-FKBP (AF488-85-FKBP), and then FLT time course was acquired after rapid (2 ms) mixing with 800 nM (final) A-CaM (AF568-26-CaM). Each graph is a representative fluorescence decay time course following mixing. The time course data were acquired in three independent experiments with similar results. Assay conditions were at 30 nM (blue) or 30 μM (red) Ca^{2+}.

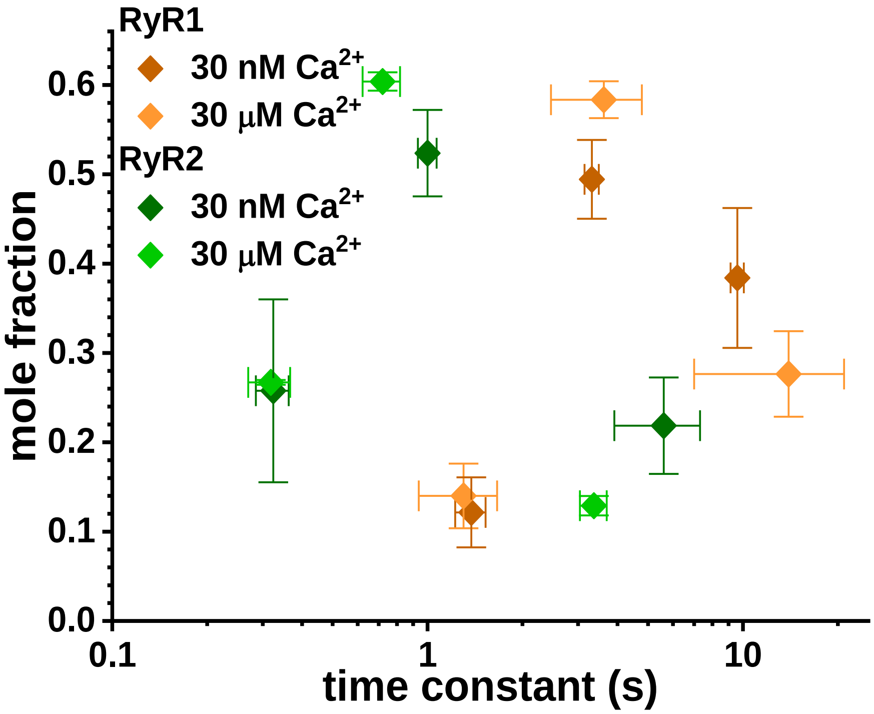

Supplementary Fig. 12 Three components for nM to μM Ca^{2+}. SR membranes from porcine skeletal (brown) or cardiac (green) muscle were labeled with D-FKBP (AF488-85-FKBP), and then FLT time course was acquired after rapid (2ms) mixing with 800 nM (final) A-CaM (AF568-26-CaM, at 30 nM Ca^{2+} (dark brown for RyR1 and dark green for RyR2) and 30 μM Ca (light brown for RyR1 and light green for RyR2) . Representative FLT time courses are shown in Supplementary Fig. 11. The FRET data fits best to three-exponential analysis. The parameters of that fit are shown as time constant vs amplitude fraction for CaM binding to RyR1 or RyR2 at 30nM or 30μM Ca^{2+}. Data shown as mean ±SD, n = 3, with individual data acquired on separate days.

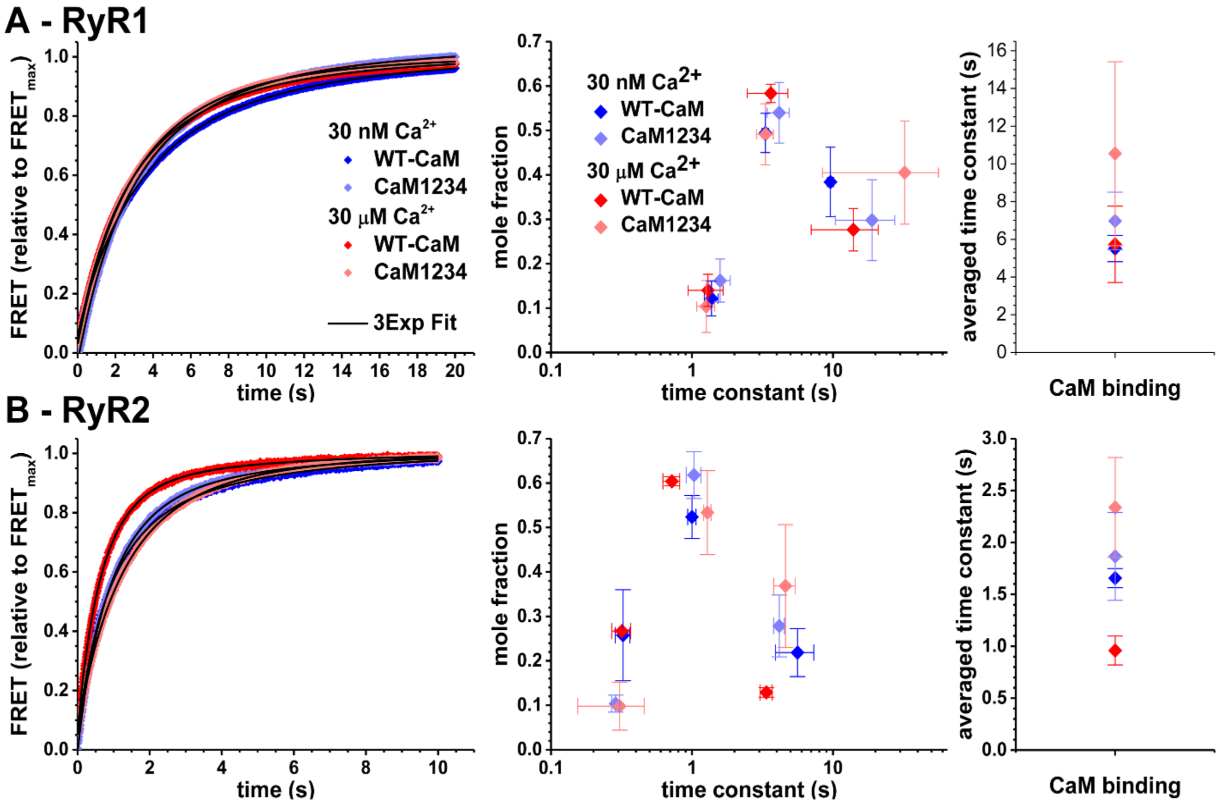

Supplementary Fig. 13 Kinetics of A-CaM binding to RyR1 and RyR2 in 30 nM and µM Ca^{2+}. SR membranes from porcine skeletal (**A**) or cardiac (**B**) muscle were labeled with D-FKBP (AF488-85-FKBP), and then FLT time course was acquired after rapid (2 ms) mixing with 800 nM (final) A-CaM (AF568-26-CaM). Representative fluorescence decay time courses shown in Supplementary Fig 10. Representative FRET time course following mixing (left panels). The FRET time courses were acquired in three independent experiments with similar results. All data was fit with three-exponential analysis, with time constant and amplitude values shown in middle panel. In right panel, amplitude-weighted average time constant values for the binding of CaM (dark) or CaM$_{1234}$ (light) to RyR1 (**A**) or RyR2 (**B**) at 30 nM (blue) or 30µM (red) Ca^{2+}. Data shown as mean ±SD, n = 3, with independent data acquired on separate days.

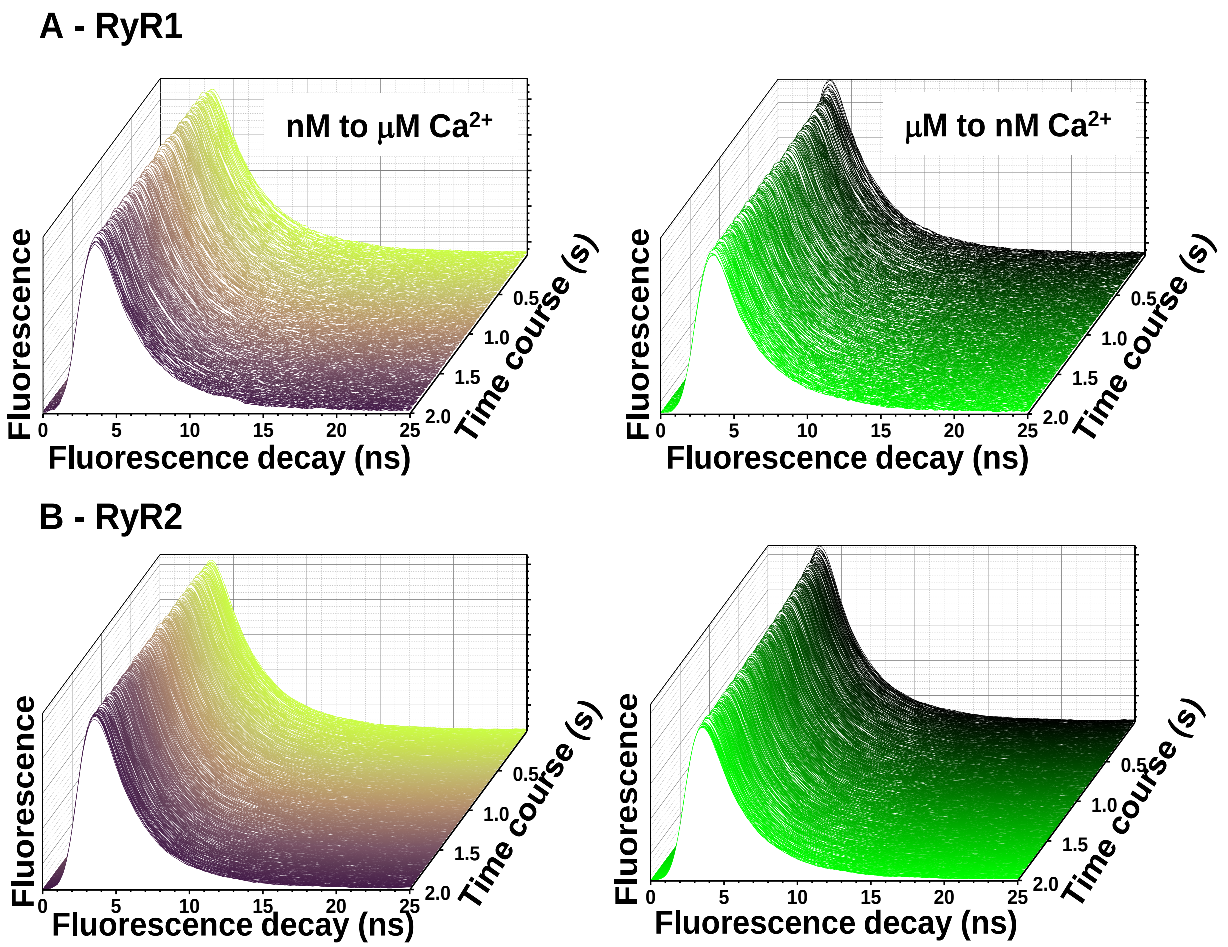

Supplementary Fig. 14 Representative fluorescence decay time course for the Ca^{2+} driven RyR-bound CaM structural transitions. SR membranes from porcine skeletal (A) or cardiac (B) muscle were labeled with D-FKBP (AF488-85-FKBP), incubated with 1.6 μM CaM and then FLT time course was acquired after rapid (2 ms) mixing with (left panel, yellow to purple) Ca^{2+} to increase [Ca^{2+}] from 30 nM to 30 μM or (right panel, dark green to light green) EGTA to reduce [Ca^{2+}] from 30 μM to 30 nM. Representative fluorescence decay time course following mixing. The data were acquired in three independent experiments with similar results.

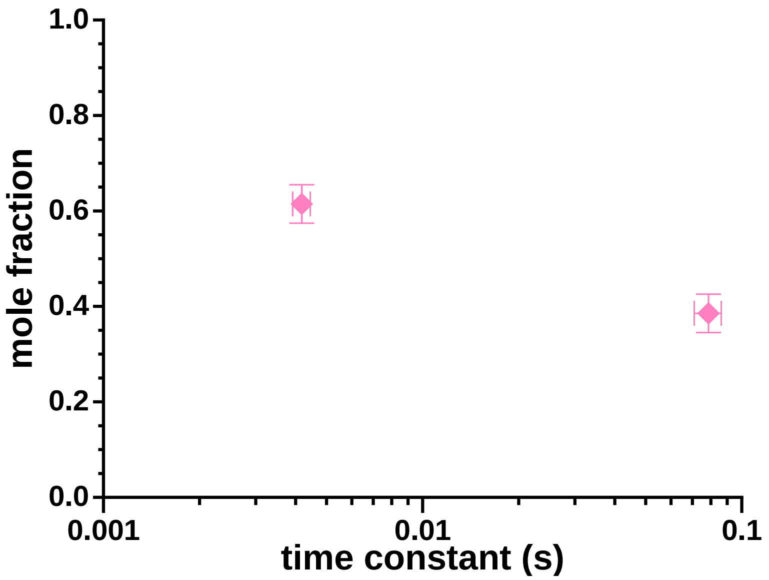

Supplementary Fig. 15 Parameters of two-exponential fits of FLT-FRET data for nM to μM Ca^{2+}-driven structural shift of CaM bound to RyR1. SR membranes from porcine skeletal muscle were labeled with D-FKBP (AF488-85-FKBP), incubated with 1.6 μM CaM and then FLT time course was acquired after rapid (2 ms) mixing with Ca^{2+} to increase [Ca^{2+}] from 30 nM to 30 μM (pink). Representative FLT waveforms are shown in Supplementary Fig 14, and representative FLT-FRET are shown in Fig. 6. Data fits best to two exponentials. Graph displays fitting parameters as time-constant and amplitude fraction. Data shown as mean ±SD, n = 3, with independent data acquired on separate days.

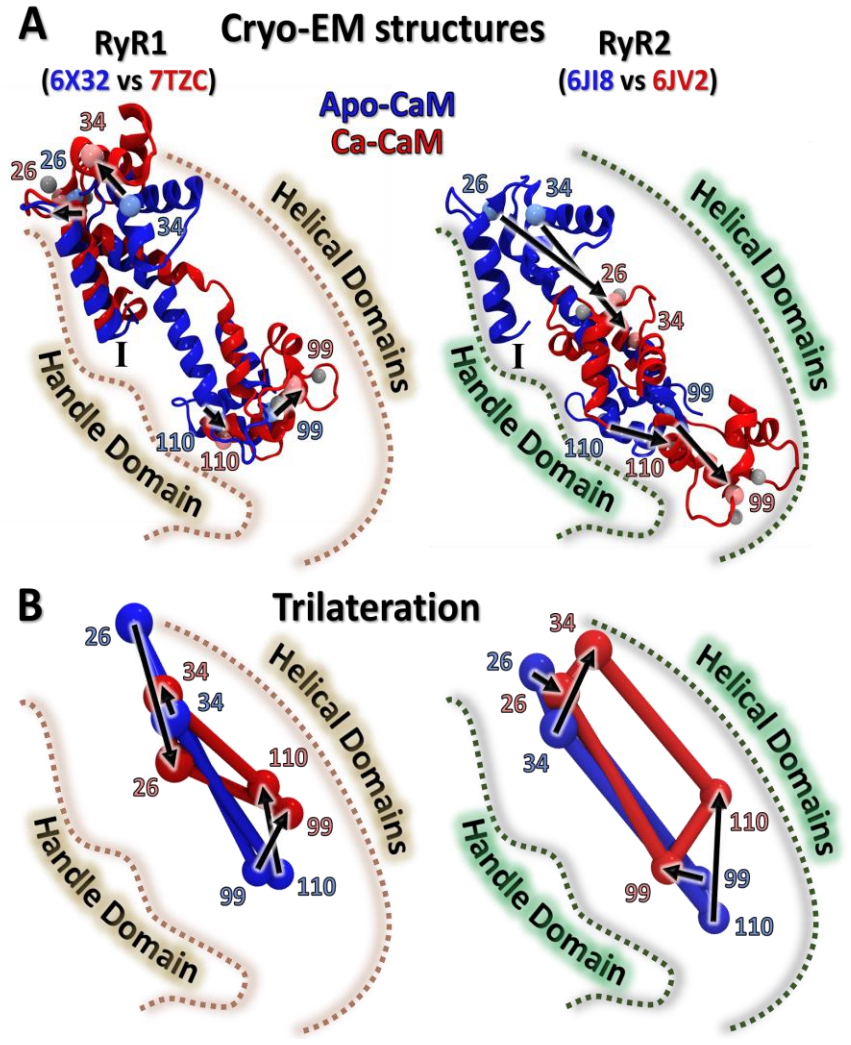

Supplementary Fig. 16 Ca^{2+} driven structural transitions of CaM on RyR1 or RyR2 from cryo-EM and trilateration. A) Cryo-EM CaM structural shifts. Apo- (blue) and Ca-CaM (red) from RyR1 structures 6X32[3] and 7TZC[13], respectively, aligned on the Handle domain indicated by dotted lines (left). Apo- (blue) and Ca-CaM (red) from RyR2 structures 6JI8 and 6JV2[4], respectively, aligned on the Handle domain indicated by dotted lines (right). Apo-CaM in both RyR1 and RyR2 have similar conformations and are bound in the same location on RyR. In RyR1 helix I of CaM does not change position upon the transition from apo-CaM to Ca-CaM. Helices II and III rotate upwards by about 45°. CaM helix IV (which connects the two lobes) has rotated following the C-lobes shift toward the RyR1 Helical Domains. For site 26, this results in no significant change in position. For site 34, a slight upward shift is observed. The C-lobe sites (99 and 110) are shifted just slightly towards the Helical Domains. In RyR2, the N-lobe is rotated about 180° and shifted much lower compared with apo-CaM. The C-lobe has undergone a rotation and a shift towards Helical Domain 2. Ca-CaM has also adopted a more compact conformation. Both the 26 and 34 positions have shifted a substantial distance to a lower position. Positions 99 and 110 have shifted a significant distance as well. **B) FRET-trilaterated CaM structural shifts.** The center of the trilaterated loci for AF568 probes bound to the indicated CaM residues are shown as spheres, drawn in blue for low Ca^{2+} (apo-CaM) and red for high Ca^{2+} (Ca-CaM). The spheres are connected by lines to better show the change in conformation and rotation as suggested by these acceptor-labeled CaM sites. In RyR1, a Ca^{2+}-induced 180° rotation, and a shift downward, are observed in the N-lobe and a shift upward is observed in the C-lobe. Site 26 has shifted substantially lower, while site 34 shifted slightly. Sites 99 and 110 have shifted upwards. This suggests that Ca-CaM has shifted downwards and adopted a more compact conformation in RyR1. In RyR2 a Ca^{2+}-induced ~90° rotation is observed in the N-lobe and a minor shift upward is observed in the C-lobe. Site 26 only undergoes a minor shift, while site 34 shifts upwards. Sites 99 and 110 are shifted upwards. This suggests that in RyR2 the shift of CaM upon Ca^{2+} binding is similar to what is observed in the RyR1 cryo-EM structures. Arrows indicate the direction of structural shifts from low to high [Ca^{2+}].
